# Altitudinal shift of malaria vectors and malaria elimination in Nepal

**DOI:** 10.1186/1475-2875-13-S1-P26

**Published:** 2014-09-22

**Authors:** Meghnath Dhimal, Bodo Ahrens, Ulrich Kuch

**Affiliations:** 1 Nepal Health Research Council (NHRC), Ministry of Health and Population Complex, Kathmandu, Nepal; 2 Institute for Atmospheric and Environmental Sciences (IAU), Goethe University, Frankfurt am Main, Germany; 3 Biodiversity and Climate Research Centre (BiK-F), Frankfurt am Main, Germany; 4 Institute of Occupational Medicine, Social Medicine and Environmental Medicine, Goethe University, Frankfurt am Main, Germany

## Background

Malaria elimination is a goal of many endemic countries with the ultimate goal of global malaria eradication. Based on the achievements the country has made over the last decade, Nepal has been preparing for a malaria pre-elimination phase since 2011 with the ambitious vision of a malaria-free Nepal by 2026. However, a number of challenges have been identified in recent studies including high rates of relapse/re-infection with *Plasmodium vivax*, persistence of *Plasmodium falciparum* and clinically suspected malaria, continuous import of malaria cases, underreporting of mixed infections (*P. falciparum* and *P. vivax*), and climate change. In this study, we report, in addition, on altitudinal shifts of malaria vectors in Nepal.

## Materials and methods

To obtain data on the present altitudinal distribution of malaria vectors, entomological surveys were carried out in eastern Nepal between 60 and 2,500 m above sea level. The surveys were conducted from September to October 2012 and April to May 2013 using aspirator and CDC light trap collection of adult mosquitoes in houses, cattle sheds and natural outdoor shelters. In addition, Anopheles larvae were collected from potential breeding sites, and geographical position and environmental data were recorded for all collection sites. The collected mosquitoes were morphologically identified in the field and then deep frozen for future molecular analyses.

## Results

Based on the findings of the morphological identification, known malaria vectors in Nepal (*Anopheles fluviatilis*, *Anopheles annularis* and *Anopheles maculatus* complex members) were recorded up to 1,820 m above sea level. This is the highest record of malaria vectors in eastern Nepal so far. Moreover, larvae of Anopheles spp. were recorded up to 2,310 m above sea level; their species-level identification is in progress. The densities of these vectors were significantly higher in the post-monsoon season (end of September to October) compared to the pre-monsoon (April-May) season.

## Conclusions

Taking into account the evidence from climate change studies and previous entomological surveys in Nepal, it can be concluded that malaria vectors have already shifted to higher altitudes of Nepal due to climatic and other environmental changes, indicating a serious health risk for mountain people and tourists. The presence of potential malaria vectors in the highland areas, lack of vector-control interventions, higher rates of warming in the mountains compared to the lowlands and the continuous introduction of malaria cases from abroad may pose challenges for achieving the goal of malaria elimination in Nepal by 2026.

